# Cerebellar Functional Lateralization From the Perspective of Clinical Neuropsychology

**DOI:** 10.3389/fpsyg.2021.775308

**Published:** 2021-12-10

**Authors:** Anna Starowicz-Filip, Katarzyna Prochwicz, Joanna Kłosowska, Adrian Andrzej Chrobak, Aneta Myszka, Barbara Bętkowska-Korpała, Borys Kwinta

**Affiliations:** ^1^Department of Medical Psychology, Jagiellonian University Medical College, Kraków, Poland; ^2^Department of Neurosurgery, University Hospital in Krakow, Kraków, Poland; ^3^Institute of Psychology, Jagiellonian University, Kraków, Poland; ^4^Department of Adult Psychiatry, Jagiellonian University Medical College, Kraków, Poland; ^5^Department of Neurosurgery, Jagiellonian University Medical College, Kraków, Poland

**Keywords:** focal cerebellar lesions, functional laterality, cognitive functions, ACE III test, neuropsychology

## Abstract

**Objective:** The cerebellar functional laterality, with its right hemisphere predominantly involved in verbal performance and the left one engaged in visuospatial processes, has strong empirical support. However, the clinical observation and single research results show that the damage to the right cerebellar hemisphere may cause extralinguistic and more global cognitive decline. The aim of our research was to assess the pattern of cognitive functioning, depending on the cerebellar lesion side, with particular emphasis on the damage to the right cerebellar hemisphere.

**Method:** The study sample consisted of 31 patients with focal cerebellar lesions and 31 controls, free of organic brain damage. The Addenbrooke’s Cognitive Examination ACE III and the Trail Making Test TMT were used to assess patients’ cognitive functioning.

**Results:** Left-sided cerebellar lesion patients scored lower than controls in attention and visuospatial domain, but not in language, fluency, and memory functions. Participants with right-sided cerebellar lesion demonstrated a general deficit of cognitive functioning, with impairments not only in language and verbal fluency subscales but also in all ACE III domains, including memory, attention, and visuospatial functions. The TMT results proved that cerebellar damage is associated with executive function impairment, regardless of the lesion side.

**Conclusion:** The cognitive profiles of patients with cerebellum lesions differ with regard to the lesion side. Left-sided cerebellar lesions are associated with selective visuospatial and attention impairments, whereas the right-sided ones may result in a more global cognitive decline, which is likely secondary to language deficiencies, associated with this lateral cerebellar injury.

## Introduction

Since the influential and significant Schmahmann and Sherman’s (1998) on *cerebellar cognitive affective syndrome* (CCAS) in patients with cerebellar lesions appeared, the role of the cerebellum in cognitive functioning has been widely explored.

Cerebellar cognitive affective syndrome was proposed to reflect a constellation of deficits in: (a) executive function (deficient set-shifting, verbal fluency, multitasking, planning, sequencing, and organizing activities); (b) visuospatial cognition (visuospatial disintegration, deficit in copying, and conceptualizing drawn figures); (c) language (agrammatism, dysnomia, and dysprosodia); and (d) emotion (flattening of affect or disinhibition, impulsive actions, overfamiliarity, regressive, and childlike behavior in some patients) (Schmahmann and Sherman, 1998).

A further research confirmed that damage limited to the cerebellum may cause deficits in several cognitive processes, such as working memory (Ziemus et al., 2007; Hautzel et al., 2009; Marvel and Desmond, 2010; Misciagna et al., 2010; Marvel et al., 2012; Baier et al., 2014), phonological short-term memory (Ph-STM) (Timmann and Daum, 2007), episodic memory (Andreasen et al., 1999; Exner et al., 2004; Fliessbach et al., 2007), attention and processing speed (Steinlin et al., 2003; Beebe et al., 2005), executive functions including planning and sequencing (Levisohn et al., 2000), visuospatial functions (Starowicz-Filip et al., 2017), and word fluency (Arasanz et al., 2012).

The cerebellar involvement in the regulation of cognitive processes is strongly supported by the neuroanatomy and neuroimage date, which provide evidence of the existence of pathways between the cerebellum and associative cortex areas, especially prefrontal and posterior parietal cortex, involved in higher cognitive functions (Schmahmann, 1996). One part of the cerebellar cortex (lobules IV, V, VI, parts of HVIIB and HVIII) received projections from the primary motor cortex, responsible for skilled motor control, and another part of the cerebellar cortex (Crus I and Crus II) received afferents from the prefrontal area, associated with higher cognitive functions (Kelly and Strick, 2003; Ramnani, 2006). In general, several neuroimaging studies show the activation of the posterior cerebellar lobe (lobule VI, lobule VII A, including Crus I and Crus II, lobule VIIB) while performing cognitive tasks (Bucker, 2013; Stoodley et al., 2016). Studies also emphasize particularly the significant role of dentate nuclei in cognition (Clower et al., 2001; Grimaldi and Manto, 2011). The mechanism of cerebellar involvement in cognitive functions is explained in different ways but the most popular is Schmahmann’s (1991) *dysmetria of thought* theory. According to this, there is a hypothesis that in the same way as the cerebellum regulates the rate, rhythm, and accuracy of movements, so it may influence the speed, capacity, and appropriateness of mental processes. Hypo- and hypermetria in the motor activation, observed in the cerebellar damage, can be extended to the non-motor domain and manifests as the *cerebellar cognitive affective syndrome* Cerebellar cognitive affective syndrome with visuospatial impairments, linguistic deficits, executive dysfunction, and affective disturbances (Schmahmann and Sherman, 1998).

Substantial studies on the role of the cerebellum in the regulation of cognitive functions focused on searching for cognitive profile patterns in individuals with right-sided and left-sided cerebellum damage. In the early 1990s, Botez-Marquard et al. (1994) described functional lateralization of cerebellum showing that a left cerebellar hemisphere lesion results predominantly in attention and visuospatial deficits. About 2 years later, Mariën et al. (1996) introduced the concept of a “lateralized linguistic cerebellum,” presenting a follow-up study of a patient with aphasia being a result of a right cerebellar infarct. The above research initiated a series of studies on functional lateralization of the cerebellum. These studies indicated that lateralized cerebellar damage leads to cognitive deficits considered as typical for contralateral cerebral lesions; that is, right cerebellar hemisphere is predominantly involved in verbal performance, whereas the left one is engaged in visuospatial processes (Fiez et al., 1992; Schmahmann and Pandya, 1997; Riva and Giorgi, 2000; Schmahmann, 2001; Scott et al., 2001; Molinari et al., 2004; Hokkanen et al., 2006; Tavano et al., 2007; Leggio et al., 2008; Baillieux et al., 2010; Stoodley et al., 2010).

This cerebellar functional laterality has a strong support in neuroanatomic and neuroimaging data. Functional neuroimaging studies by means of a single-photon emission computerized tomography (SPECT) (Baillieux et al., 2006; Marian et al., 2007) and functional magnetic resonance imaging (fMRI) (Fink et al., 2000; Jansen et al., 2005) have convincingly shown the importance of the contralateral cerebello-cerebral network in cognitive modulation, opposite to motor deficits which are ipsilateral to cerebellar hemisphere lesions (Riva and Giorgi, 2000). The majority of streamlines passing through the superior cerebellar peduncle connect the cerebellar hemispheres with contralateral cortical associative areas through the ventrolateral thalamus (Bernard et al., 2012).

Although most of the studies support functional lateralization hypothesis, many of them are based on the very small study samples (Scott et al., 2001; Baillieux et al., 2010). There is also a growing body of research which did not confirm the cerebellar crossed laterality effect (Levisohn et al., 2000; Beebe et al., 2005). Moreover, although single scientific reports confirmed the clear effect of cerebellar functional lateralization of left cerebellar hemisphere, and its connection with attention and visuospatial dysfunctions, the cognitive functioning of patients with cerebellar right-sided lesions shows the tendency to more global decline, going beyond the selective damage to linguistic functions (Baillieux et al., 2009; Daszkiewicz et al., 2009; Starowicz-Filip et al., 2020).

According to the above-mentioned research, the main goal of our study was to determine the pattern of cognitive functioning of the patients with cerebellar damage in dependence on the lesion side. Based on the available studies and neuroanatomical data (Häberling and Corballis, 2016; Kavaklioglu et al., 2017; Guell et al., 2018), we hypothesized that patients with left-sided cerebellar lesions may be characterized predominantly by weaker visuospatial and attention functions according to control group. Regarding patients with right-sided cerebellar lesion, we intended to explore whether they are characterized by selective language deficits or by more global cognitive decline.

## Materials and Methods

### Participants

#### The Cerebellar Lesion Group

The cerebellar lesion group was composed of 31 participants (17 women, 14 men; 55 and 45%, respectively) with mean age of 51.51 years (SD = 16.93, age range: 20–76).

Patients were recruited prospectively from the neurosurgery ward, rehabilitation clinic, and the outpatient care. The inclusion criteria for the cerebellar lesion group were focal cerebellar damage (tumor, stroke or vascular malformation, information based on MRI results, histopathology results, and neurosurgeon or neurologist qualification and opinion), whereas the exclusion criteria were the presence of extracerebellar brain damage and/or psychiatric illness. The cerebellar lesion location was determined by the radiologist, based on MRI results. Participants were diagnosed with left-sided cerebellar lesions (13 participants), right-sided cerebellar lesions (15 participants), and isolated vermis damage (three participants). Out of 31 patients, 18 were diagnosed with cerebellar tumor, seven with cerebellar stroke, two with posttraumatic hematoma, and four with cerebellar arteriovenous malformation (AVM). Patients with extracerebellar pathology seen in an MRI examination were not included in the study. Among patients with cerebellar tumors, there were seven patients with metastatic tumors, five patients with astrocytoma WHO 1, two patients with meningioma WHO 2, two patients with medulloblastoma WHO 4, one patient with glioblastoma multiforme WHO 4, and one patient with ependymoma WHO 1. None of the cerebellar patients had cerebellar atrophy.

The neuropsychological examination was performed after the resection of the tumor. The mean time from cerebellar tumor resection or stroke was 4.27 months. Among the cerebellar lesion, three patients had hydrocephalus and needed shunting. None of the patients had hearing difficulties. Only two patients had visual problems.

#### The Control Group

The control group was composed of 31 subjects (11 women, 20 men; 35 and 65%, respectively) with mean age of 57.67 years (SD = 13.26, age range: 24–83) diagnosed with a spine degenerative disease. The exclusion criteria were history of neurological and/or psychiatric illness. These participants were recruited from the Rehabilitation Center and Neurosurgery Department of University Hospital. The control subjects were matched with patients with cerebellar damage with regard to age, sex, and education level. They were also exposed to stress related to treatment, the need to visit a doctor, and hospitalization, in some ways similar to stress accompanying the experimental group of cerebellar patients. This selection procedure allows us to control the possible distorting influence of external stress on the results of cognitive tests.

The same study participants were included in our publication on the usefulness of the ACE III test in assessing cognitive processes of patients with cerebellar damage (Starowicz-Filip et al., 2021).

All participants signed an informed written consent to the assessment in line with the Declaration of Helsinki. The study was approved by the local Ethics Committee. No financial remuneration was offered as an incentive to participate.

The detailed sociodemographic characteristics of the sample are presented in Table 1.

**TABLE 1:** Sociodemographic characteristics of the sample.

Variable	Statistic	Left-sided cerebellar lesion (*N* = 13)	Right-sided cerebellar lesion (*N* = 15)	Controls (*N* = 31)	χ^2^ (df)	*p*
Age	Mean (SD)	50.089 (18.464)	53.200 (16.917)	57.670 (13.262)	1.466 (2)	0.481
Time since lesion (days)	Mean (SD)	122.769 (175.061)	128.800 (92.139)	-	2.127 (1)	0.145
Sex	*N* (%)				3.762 (2)	0.152
Women		8 (61.538)	9 (60.000)	11 (35.484)		
Men		5 (38.462)	6 (40.000)	20 (64.516)		
Education	N (%)				4.127 (2)	0.660
Primary school		1 (7.692)	1 (6.667)	2 (6.452)		
Secondary school		3 (23.077)	5 (33.333)	10 (32.258)		
High school		6 (46.154)	2 (13.333)	9 (29.032)		
University		3 (23.077)	7 (46.667)	10 (32.258)		

*Three participants with vermis damage were excluded from the analyses; to check whether there are significant differences between groups on “age” and “time since lesion” variables, Kruskal–Wallis and Mann–Whitney tests were used; to check whether there is significant association between “sex,” “education,” and “group” variables, chi-squared tests were utilized.*

### Instruments

#### The Addenbrooke’s Cognitive Examination III

The Addenbrooke’s Cognitive Examination ACE III (Hodges and Larner, 2017) is a screening instrument used to establish cognitive impairments in five cognitive domains: attention, memory, language, verbal fluency, and visuospatial abilities. (1) Attention domain (18 points) is tested by asking the patient about the date and current location, repeating back three words and serial subtraction (Bruno and Schurmann Vignaga, 2019). (2) Memory domain (26 points) is assessed by asking the patient to recall three previously repeated words, coding and recalling the name and address of a person and recalling widely known surnames from the historical facts or from the politics. (3) Fluency domain (14 points) is tested by asking the patient to list as many words as he or she can think of, starting with a specific letter, within 1 min (phonemic fluency), or naming as many animals, as he or she can in 1 min (semantic fluency). (4) Language domain (26 points) is examined by assessing speech comprehension: the patient is asked to complete a set of commands, such as “place the paper on the pencil,” graphomotor abilities (by writing two grammatically complete sentences), repetition and articulation (by repeating several polysyllabic words; confrontational naming is tested by naming objects shown in 12 drawings and by answering contextual questions about some of the objects), and lexia (by reading words with irregular sound-spelling correspondence). (5) Visuospatial functions (16 points) are tested by asking the patient to copy two diagrams (cubes), to draw a clock face with the hands set at a specified time, to count a set of dots and to recognize four fragmented letters (visual gnosis) (Bruno and Schurmann Vignaga, 2019).

The total ACE III score is based on the maximum score of 100, with higher scores indicating better cognitive functioning. Although the ACE III is a brief screening tool originally designed to assess dementia in a population of elderly people (Mathuranath et al., 2000), its applicability in the detection of cognitive impairments has already been proved in the population of younger people, with other health conditions, such as stroke (Lees et al., 2017), multiple sclerosis (Figlus et al., 2018), and brain tumors (Kerrigan et al., 2011; Cherkil et al., 2017). Recent study has proved its good sensitivity and diagnostic specificity in the assessment of cognitive dysfunctions of patients with cerebellar lesions (Starowicz-Filip et al., 2021).

#### The Trail Making Test

The TMT (Reitan and Wolfson, 1993) is a useful assessment tool to evaluate a wide range of cognitive processes including attention, visual search and scanning, sequencing and shifting, psychomotor speed, abstraction, flexibility, and ability to modify a plan of action. It is perceived as a good screening tool for assessing executive functions (Lezak et al., 2004). The TMT is divided into trials A and B: in condition A, the participant’s task is to connect the given numbers in a numerical sequence (i.e., 1-2-3, etc.) as rapidly as possible by drawing a line linking subsequent numbers; in condition B, the participant’s task is to draw lines to connect numbers and letters in an alternating numeric and alphabetic sequence (i.e., 1-A-2-B, etc.) as rapidly as possible. Both parts of TMT, Trail A and Trail B, are thought to assess attention capacity, psychomotor speed, and sequencing abilities. However, Trail B is thought to assess set-shifting which requires individuals to switch their attention between two rules or tasks (Miyake et al., 2000).

### Procedure

All participants were tested individually by a clinical neuropsychologist during a single session lasting about 40 min. Before attending, the study participants were informed about the possibility of stopping the testing or taking a break at any time during the research.

### Statistical Analysis

Empirical distribution of continuous variables was described using median and quartiles. The comparison of groups was made using the Kruskal–Wallis test and *post hoc* Dunn’s test with the Bonferroni–Holm correction. We decided not to use the overly conservative Bonferroni method because this kind of correction may increase the probability of false negatives (not detecting the difference between lesion patients and controls where there is one) which should be avoided in a clinical setting. Therefore, we used the Bonferroni–Holm correction which seems more appropriate in this context.

All statistical analyses were performed using SPSS version 26.

## Results

To show the difference in cognitive functioning between patients with right-sided cerebellar lesions, patients with left-sided cerebellar lesions, and controls, we performed the Kruskal–Wallis test and *post hoc* comparisons between these three groups. The results are shown in Table 2.

**TABLE 2:** Comparison in screening test scores between patients with left-sided cerebellar lesions, patients with right-sided cerebellar lesions and controls (Kruskal–Wallis test).

Screening test	Left-sided cerebellar lesion Median (1st quartile; 3rd quartile)	Right-sided cerebellar lesion Median (1st quartile; 3rd quartile)	Controls Median (1st quartile; 3rd quartile)	χ^2^	*p*	ε^2^
ACE III total score	81.50 (74.00; 91.00)	84.00 (74.00; 86.00)	94.00 (86.00; 98.00)	13.63	**0.001**	0.215
ACE III attention	16.50 (14.50; 17.00)	16.00 (16.00; 17.00)	17.00 (17.00; 18.00)	9.23	**0.010**	0.134
ACE III memory	19.00 (14.00; 24.50)	23.00 (16.00; 24.00)	23.00 (20.00; 25.00)	3.97	0.137	0.036
ACE III fluency	8.00 (5.50; 11.00)	9.00 (5.00; 10.00)	12.00 (9.00; 14.00)	11.44	**0.003**	0.175
ACE III language	26.00 (22.00; 26.00)	25.00 (23.00; 25.00)	26.00 (26.00; 26.00)	10.63	**0.005**	0.160
ACE III visuospatial functions	16.00 (10.50; 16.00)	14.50 (11.00; 16.00)	16.00 (16.00; 16.00)	13.59	**0.001**	0.215
TMT A time	57.00 (52.50; 84.50)	84.50 (50.00; 106.00)	46.00 (36.00; 58.00)	12.78	**0.002**	0.200
TMT A mistakes	0.00 (0.00; 0.50)	0.00 (0.00; 1.00)	0.00 (0.00; 0.00)	3.14	0.208	0.021
TMT B time	154.00 (100.50; 225.00)	164.00 (113.00; 308.00)	89.00 (65.00; 110.00)	16.28	**0.000**	0.264
TMT B mistakes	1.00 (1.00; 5.50)	1.00 (0.00; 2.00)	0.00 (0.00; 0.00)	15.61	**0.000**	0.252

*df = 2. Statistically significant results are bolded.*

A detailed analysis revealed the presence of significant differences between left-sided cerebellar lesion group, right-sided cerebellar lesion group, and control group in the ACE III total scores and in most of the ACE III single cognitive domains, except the memory domain.

According to the TMT results, these three groups differed significantly in time of performing part A and part B of the test, and also in the number of mistakes made on the TMT part B. There was no significant difference in the number of mistakes in the TMT part A between the three groups. The pairwise comparisons between scores of patients with left-sided cerebellar lesions, patients with right-sided cerebellar lesions, and controls are shown in Table 3.

**TABLE 3:** Pairwise comparisons between scores of patients with left-sided cerebellar lesions, patients with right-sided cerebellar lesions, and controls.

Screening test	Left-sided cerebellar lesion vs. right cerebellar lesion		Left-sided cerebellar lesion vs. controls		Right-sided cerebellar lesion vs. controls	
	Standardized test statistic	*p*	Standardized test statistic	*p*	Standardized test statistic	*p*
ACE III total score	0.122	0.903	−2.810	**0.010**	−3.117	**0.006**
ACE III attention	0.067	0.946	−2.335	**0.040**	−2.548	**0.033**
ACE III fluency	0.549	0.583	−2.719	0.060	−3.066	**0.006**
ACE III language	1.592	0.222	−1.306	0.222	−3.244	**0.003**
ACE III visuospatial functions	0.639	0.523	−2.442	**0.030**	3.359	**0.003**
TMT A time	−0.954	0.340	2.101	0.072	3.384	**0.003**
TMT B time	−0.704	0,481	2.710	**0.014**	3.642	**0.003**
TMT B mistakes	1.180	0.238	3.704	**0.003**	2.382	**0.034**

*Dunn’s multiple comparison tests with the Bonferroni–Holm correction were carried out. Statistically significant results are bolded.*

The pairwise comparison indicated that patients with right-sided cerebellar lesion scored significantly lower than controls on the ACE III (total score); they also obtained lower scores on nearly all the ACE III subscales measuring individual cognitive domains, except for the memory domain. The ACE III cognitive profile of patients with left-sided cerebellar lesion appeared to be more selective with significantly lower results in the ACE III (total score) and the ACE III attention and visuospatial domain, compared with the results of the control group.

Both the right-sided and the left-sided cerebellar lesion groups made more errors on the TMT part B and needed more time to accomplish this part of the TMT test than controls. Moreover, patients with right-sided lesion needed more time than controls to accomplish part A of the TMT. The right-sided and left-sided cerebellar lesion groups did not differ in any of the ACE III and TMT scores.

The results obtained by participants in the memory (memory retrieval and memory recognition) and fluency (perseverations and intrusions) domains have been analyzed in more detail to establish whether the cognitive profile of patients with cerebellar lesions can be characterized as frontal-like, as it has been demonstrated in the previous research (Starowicz-Filip et al., 2020). Moreover, because right cerebellar lesions are linked with language impairments, we also checked which components of the ACE III language domain are mostly attenuated due to cerebellar damage. Hence, we performed the comparisons between the right-sided cerebellar lesion group, the left-sided cerebellar lesion group, and controls regarding the ACE III memory, fluency, and language subscales. The results are shown in Table 4.

**TABLE 4:** Comparison in the ACE III subscale scores of memory, fluency, and language domains between patients with left-sided cerebellar lesions, patients with right-sided cerebellar lesions, and controls (Kruskal–Wallis test).

ACE III subscales	Left-sided cerebellar lesion median (1st quartile; 3rd quartile)	Right-sided cerebellar lesion median (1st quartile; 3rd quartile)	Controls median (1st quartile; 3rd quartile)	χ^2^	*p*	ε^2^
Memory retrieval	4.00 (1.50; 6.00)	4.00 (1.00; 6.00)	6.00 (5.00; 7.00)	9.10	**0.011**	0.131
Memory recognition	5.00 (3.50; 5.00)	5.00 (5.00; 5.00)	5.00 (4.00; 5.00)	0.91	0.632	0.020
Fluency perseveration	0.00 (0.00; 0.50)	0.00 (0.00; 1.00)	0.00 (0.00; 0.00)	0.76	0.684	0.023
Fluency intrusions	1.00 (0.00; 1.00)	0.00 (0.00; 1.00)	0.00 (0.00; 0.00)	14.28	**0.001**	0.227
Language comprehension	3.00 (3.00; 3.00)	3.00 (3.00; 3.00)	3.00 (3.00; 3.00)	1.370	0.504	0.012
Language articulation	2.00 (2.00; 2.00)	2.00 (1.00; 2.00)	2.00 (2.00; 2.00)	6.064	**0.048**	0.075
Language-confrontational naming	12.00 (10.00; 12.00)	8.50 (8.00; 11.25)	12.00 (12.00; 12.00)	19.794	**0.000**	0.330
Language graphia	2.00 (2.00; 2.00)	2.00 (2.00; 2.25)	2.00 (2.00; 2.00)	1.003	0.606	0.018
Language lexia	1.00 (1.00; 1.00)	1.00 (1.00; 1.00)	1.00 (1.00; 1.00)	4.864	0.088	0.053
Language repetition	2.00 (2.00; 2.00)	2.00 (2.00; 2.00)	2.00 (2.00; 2.00)	1.169	0.557	0.015

*df = 2. Statistically significant results are bolded.*

Regarding the ACE III memory domain, the detailed analysis revealed a significant difference among the left-sided cerebellar lesion group, the right-sided cerebellar lesion group, and the control group only in the memory retrieval subscale. There were no between-group differences in the memory recognition subscale.

According to the ACE III fluency domain, the analysis showed that the three groups differed significantly only in the number of intrusions during the fluency trials. There was no difference in the number of perseverations.

As for the ACE III language subscales, significant between-group differences were observed in the scores of articulation and confrontational naming, however, not in comprehension, graphia, lexia, and repetition scores. The pairwise comparisons of scores provided by the three groups of participants are shown in Table 5.

**TABLE 5:** Pairwise comparisons in memory, fluency, and language subscale scores between patients with left-sided cerebellar lesions, patients with right-sided cerebellar lesions, and controls.

ACE III subscales	Left-sided cerebellar lesion vs. right-sided cerebellar lesion		Left-sided cerebellar lesion vs. controls		Right-sided cerebellar lesion vs. controls	
	Standardized test statistic	*p*	Standardized test statistic	*p*	Standardized test statistic	*p*
Memory retrieval	0.245	0.807	−2.199	0.056	−2.621	**0.027**
Fluency intrusions	2.180	0.058	3,773	**0.003**	1.321	0.187
Articulation	0.964	0.482	−1.172	0.482	−2.415	**0.048**
Confrontational naming	2.287	**0.044**	−1.507	0.132	−4.446	**0.003**

*Dunn’s multiple comparison tests with the Bonferroni–Holm correction were carried out. Statistically significant results are bolded.*

The pairwise comparison indicated that patients with right-sided cerebellar lesion scored significantly lower than controls on the ACE III memory retrieval, language-articulation, and language-confrontational naming subscales. The left-sided cerebellar lesion group made significantly more intrusion mistakes during the fluency trial compared with controls. Patients with left-sided lesion did not differ from the controls regarding language and memory subscales. Both groups with cerebellar lesion differed from each other only in the ACE III confrontational naming; the right-sided cerebellar lesion group obtained significantly lower results in this subtest than the left-sided cerebellar lesion group.

## Discussion

In this study, we examined the cognitive functioning of patients with cerebellar damage based on their performance in neuropsychological tasks. In particular, we examined whether the cerebellum lesion localization influences the pattern of cognitive impairments demonstrated by patients.

The results of our study revealed that individuals with focal cerebellar lesions after tumor surgery or stroke scored significantly lower than healthy controls in all investigated cognitive domains. They revealed the CCAS, which is considered as constellation of mild deficits in cognitive domains of executive function, language, spatial cognition, and emotions (Schmahmann and Sherman, 1998; Levisohn et al., 2000; Schmahmann, 2001, 2010).

Our study proved the previously described functional lateralization of the cerebellum (Riva and Giorgi, 2000; Scott et al., 2001; Hubrich-Ungureanu et al., 2002; Gottwald et al., 2004; Hokkanen et al., 2006; Krienen and Buckner, 2009; Habas et al., 2011; Starowicz-Filip et al., 2017), but only in the left-sided cerebellar lesion group. As we predicted, patients with left-sided cerebellar lesions scored lower than controls in the attention and visuospatial domains of the ACE III, and they did not differ from the control group in terms of language, fluency, and memory functioning. Regarding the right-sided cerebellar lesion group, the laterality effect did not fully reveal in its pure form. Admittedly, participants with right-sided cerebellar lesion scored significantly lower than controls in language and verbal fluency subscales of the ACE III; however, they also obtained lower scores in almost every other ACE III domain, including memory, attention, and visuospatial functions; the last one was previously assigned to left-sided cerebellar impairments. Hence, it may be assumed that they demonstrated a general deficit of cognitive functioning rather than impairment in particular cognitive domains. Moreover, in our study individuals with lesions localized in the right cerebellum hemisphere needed more time to complete both A and B parts of the TMT which probably reflects slowness of searching the visual field in this group of patients. It should be noted, however, that performing the TMT appeared to be demanding for both cerebellar lesion groups, all patients with cerebellar lesions committed more errors in the TMT part B than controls and needed significantly more time to accomplish this part of the TMT. This finding may suggest that cerebellar damage is associated with executive functions impairment regardless of lesion side.

In our study, the patients with lesions localized in the left cerebellar hemisphere showed difficulties in copying geometric shapes, Mobius band, and cube or in the clock task. Previous research on adults (Riva and Giorgi, 2000; Molinari et al., 2004; Baillieux et al., 2010; Starowicz-Filip et al., 2020) and pediatric samples (Starowicz-Filip et al., 2017) have also yielded such selective attention and visuospatial impairments against the background of preserved language functions. These visuospatial impairments appeared particularly evident in the tasks of processing complex figures mentally, such as the mental rotation task and manipulation of tridimensional stimuli (Molinari et al., 2004) which are more demanding than straightforward redrawing of visually presented objects. Contrarily, our research patients with left-sided cerebellum lesions revealed difficulties in completing relatively effortless tasks involving simple visuospatial processes, such as copying geometric shapes and drawing a clock face.

Although the disturbances of the visuospatial functions in the cerebellar left-sided lesion group clearly stood out against the background of other preserved cognitive functions, especially linguistic ones, our study did not reaffirm the previously observed cerebellar intergroup difference in visuospatial functioning. The visuospatial domain scores of patients with the left-sided lesion were not significantly lower than the scores of the right-sided lesion group; the latter one was also characterized by impairments in the spatial task when compared to controls.

Our research confirmed the well-documented effect of the right-sided cerebellar injuries on language functions and the absence of such impact in case of left-sided damage (Riva and Giorgi, 2000; Molinari et al., 2004; Baillieux et al., 2010; Lesage et al., 2017). However, it is worth mentioning that this effect was not selective, and patients with damage in the right cerebellar hemisphere were accompanied by disturbances in the extralinguistic, cognitive domains, measured with the ACE III and TMT. The more detailed analysis of patients’ language abilities, measured with the ACE III, revealed deterioration of the confrontational naming in the group with the right-sided cerebellar hemisphere damage (compared with controls and left-sided cerebellar lesion group). Moreover, right-sided patients obtained the worst results in the verbal fluency subscale of the ACE III; however, the impaired verbal fluency domain may be considered as caused by slowed down access to words in the lexical memory storage. Considerably, lower speed of name retrieval in response to specific visual stimuli was also observed by Stoodley and Schmahmann (2009) in a group of patients with neurodegenerative cerebellum damage. Petersen et al. (1989) provided a systematic account of the hemodynamic correlates of single-word processing, showing that the lexical access yielded a response of lateral parts of the right cerebellar hemisphere. Although in none of our right-hemispheric cerebellar patients, the language problems were severe enough to be described as aphasic, the literature provides examples of the so-called cerebellar aphasia after damage of the right cerebellum (Fiez et al., 1992; Mariën et al., 1996). Verbal problems in the described patients, such as impaired spontaneous speech, problems with its initiation, the presence of fragmentary sentences, severe difficulties in word generativity, and deficits in reading and writing, were analogous to those observed in transcortical motor aphasia (TCMA) (Mariën et al., 1996) or amnestic aphasia (Fiez et al., 1992). Cerebellar aphasia has also been reported by other authors (Cook et al., 2004; Baillieux et al., 2010; Bartczak et al., 2011; Blancart et al., 2011; De Smet et al., 2011), who studied not only individual cases but also groups of patients (Karaci et al., 2008). Our results that show lower fluency scores (measured as the sum of responses in the examination of semantic and phonological fluency) in the right-sided cerebellar lesion group are also in line with the previous research (Leggio et al., 1995; Hubrich-Ungureanu et al., 2002; Richter et al., 2007; O’Hare et al., 2008), emphasizing the role of the right cerebellar hemisphere in the regulation of fluency function. Hubrich-Ungureanu et al. (2002), using fMRI during a silent verbal fluency task in healthy volunteers, showed brain activation in the left frontoparietal region and simultaneously in the contralateral right cerebellar hemisphere among the right-handed subjects. Furthermore, a reverse activation pattern that engaged the right cerebral cortex and the left cerebellar hemisphere was observed in left-handed subjects with atypical right hemisphere dominance for language.

With regard to executive functions, our research roughly confirmed the hypothesis of the bilateral cerebellar representation of these cognitive processes (Klein et al., 2016). In our study, both the right-sided and the left-sided cerebellar lesion groups committed more mistakes in part B of the TMT than controls; moreover, they also needed more time to accomplish this part of the test. This suggests that cerebellar lesions might in general influence the effectiveness of spatial working memory and mental abilities to shift attention in the TMT. Therefore, our results are in line with previous research emphasizing the executive function impairments as an effect of cerebellar lesions (Grafman et al., 1992; Karatekin et al., 2000; Levisohn et al., 2000; Riva and Giorgi, 2000; Steinlin et al., 2003; Beebe et al., 2005; Bauer et al., 2009; Riva et al., 2013; Mak et al., 2016; Salman and Tsai, 2016). However, in this study, only the right-sided cerebellar lesion group needed significantly more time than controls to accomplish part A of the TMT, which suggests a more global slowdown in information processing and visual field searching than just a selective executive deficit, present in this group. However, the observation that reduced speed of information processing yielded only in the case of patients with right-sided cerebellar injuries, whereas not in those with left-sided lesions, is contrary to the findings of other studies which emphasized higher response times in the Stroop Color and Word Test (SCWT) and the TMT parts A and B in both the right and left cerebellar hemispheric groups (Mak et al., 2016). Notwithstanding, more detailed examination indicated that in our study, a subtle effect of cerebellar lateralization in the scope of executive functions was also visible in the case of patients with damage localized in the left cerebellar hemisphere. Whereas quantitatively this group did not differ from controls in terms of verbal fluency, these patients made significantly more intrusion mistakes. This difference was not present in the right-sided group and controls. The intrusion mistakes may be considered as an expression of a less effective access strategy to lexical and semantic memory stores to make correct selections, and also reduced ability to inhibit the incorrect verbal associations (Tröster et al., 1998). Both these executive processes required keeping a constant level of focused attention. It is the weakened attention processes, visible in our research in patients with left-sided cerebellar damage that could explain the increased number of intrusion errors in the fluency examination. A greater severity of intrusion errors in cognitive tasks including verbal fluency has so far been reported among patients with frontotemporal dementia (Rouleau et al., 2001) or subcortical degenerations (Perez et al., 2020) but not in patients with cerebellar damage. However, from the functional point of view, it can be anticipated that the cerebellum, through its connections with the subcortical nuclei, could participate in the regulation of the inhibition networks in the brain and is involved in the detection and correction of errors (Falkenstein et al., 2001; Ullsperger and von Cramon, 2006; Ito, 2008; Brunamonti et al., 2014). This hypothesis has already been confirmed in the functional neuroimaging studies on healthy individuals during the SCWT (Egner and Hirsch, 2005; Stoodley et al., 2010).

Regarding the memory domain, this study revealed a pattern characterized with difficulties in recalling previously encoded information from the long-term memory storage and relatively well-preserved recognition. The recalling difficulties are also characterized by cerebellar laterality, as in our research, they concern only patients with right-sided cerebellar hemispheric lesions. In general, our research confirms the conclusions reached in previous single studies about the frontal-like nature of memory disorders accompanying patients with cerebellar damage (Koziol et al., 2014; Starowicz-Filip et al., 2020). However, contrarily to present findings, the frontal-like profile of cerebellar memory impairments obtained for pediatric patients in Starowicz-Filip et al.’s (2020) was irrespective of the cerebellar lesion side. What is more, the pattern of cognitive dysfunction yielded in the current research for patients with cerebellar damage suggests that their memory problems may be mostly secondary to executive dysfunctions, such as invalid planning and organization. The difficulty in retrieval from long-term memory storage might be due to primary acquisition organization insufficiency. It is worth mentioning that executive function impairments (significant planning and organization problems) as an effect of cerebellar lesions have been described in many previous studies (Grafman et al., 1992; Karatekin et al., 2000; Levisohn et al., 2000; Riva and Giorgi, 2000; Steinlin et al., 2003; Beebe et al., 2005; Bauer et al., 2009; Riva et al., 2013; Mak et al., 2016; Salman and Tsai, 2016).

Our findings show that the cognitive profile of patients with right-sided cerebellar lesions is characterized by a generally lower level of cognitive performance with weaker language, visuospatial, attention and memory functions, and slowness of information processing which is quite novel, and we could not find similar effects regarding functional cerebellar lateralization in any other research on adult populations with cerebellar damage. However, when analyzing the research on children with damage to the cerebellum, a similar imbalance in intellectual functioning and lower intelligence of children with right-sided cerebellar lesion was observed in Daszkiewicz et al.’s (2009). Also, Baillieux et al. (2009) demonstrated that cerebellar right-sided lesions are accompanied with greater impairment not only in linguistic processing, but also in logical reasoning, whereas left-sided damage is related to deficits in attention, visuospatial functioning, non-verbal problem-solving, and mimicking typical right hemisphere dysfunctions. Steinlin et al. (2003), however, obtained opposite findings showing that children with left-sided cerebellar tumors are more behaviorally and neuropsychologically affected than those with tumor localized on the right side of the cerebellum; this effect, however, could be explained by a more pronounced vermis involvement in the former group (Steinlin et al., 2003).

The selective visuospatial and attention impairments related to the left cerebellar hemisphere lesions can be explained by the crossconnections of this cerebellar hemisphere with the opposite cortical associative areas of the right cerebral hemisphere, especially the parietal lobe (Fink et al., 2000), whereas the more global, extralinguistic deficits revealed by patients with the right cerebellar hemisphere damage may underlie a possible deterioration of the extralinguistic cognitive domains, which is secondary to language abnormalities. These language abnormalities present in the patients with right-sided cerebellar lesions are based on neuroanatomical connections between the right cerebellar hemisphere (their lateral part) and contralateral Brodmann’s areas 6, 44, and 45 *via* the nucleus ventralis intermedius and nucleus ventralis anterior of the thalamus (Engelborghs et al., 1998) crucial for language modulation. A large body of evidence yielded that the posterior lateral right hemisphere (lobules VI, Crus I, and II) of the cerebellum is associated with phonological and semantic processing (Frings et al., 2006; Stoodley and Schmahmann, 2009).

It is likely that impairment of verbal skills caused by the damage of the right cerebellum hemisphere may in turn affect the scores of non-verbal tasks through dysregulation of the so-called inner speech which organizes and controls a given activity. The strong involvement of the cerebellum in the inner speech mechanism was proved in the research of Marvel and Desmond (2012). “Internal” covert speech of word strings (silent, prearticulatory, but fully parsed representation of the sound structure of verbal utterances) elicits a right cerebellar hemisphere response, concomitant with a hemodynamic reaction of the contralateral lower precentral gyrus (Ackermann et al., 2007). The computational power of the cerebellum subserves the temporal organization of the sound structure of utterances even at a prearticulatory level in terms of the adjustment of syllable lengths, including the “speaking rate” of the internal verbal code and the implementation of anticipatory coarticulation effects (Ackermann et al., 2004; Ben-Yehudah et al., 2007). Thus, the cerebellar impairments may compromise cognitive operations dependent upon inner speech such as the subvocal rehearsal system of verbal working memory or may disturb the linguistic support of executive functions (Ackermann et al., 2007). In consequence, the “internal speech” mechanisms may be involved in a variety of executive functions such as working memory, solving mathematical equations or strategical planning, and frontal lobe-dependent learning tasks (Ackermann et al., 2007). The fMRI studies strongly support the role of the right cerebellar hemisphere in verbal working memory. The superior cerebellum, lobules VI, and Crus I may initiate an internal motor sequence of phonological content during information encoding, whereas the inferior cerebellar, lobules VIIB/VIII, may support phonological storage during the maintenance of verbal information (Mariën et al., 2014). The subvocal rehearsal mechanism of VWM appears to involve the right cerebellum, apart from the left inferior frontal gyrus, supplementary motor area, and insula (Paulesu et al., 1993).

Ben-Yehudah et al. (2007) listed three theoretical mechanisms that may explain the role of the cerebellum in verbal working memory. First, the cerebellum takes part in an articulatory rehearsal mechanism in phonological loop. The second explanation is error-driven adjustment model. According to this model, the cerebellum is the one important component of this monitoring process that detects errors and adjusts the planned articulation prior to its execution (Ben-Yehudah et al., 2007), and therefore is involved in both the fluency and the absence of errors in speech. The last model concluded that the cerebellum is important for the temporal organization of internal speech (Ackermann et al., 2004, in Ben-Yehudah et al., 2007). Recently, the results of Ailion et al.’s (2020) have provided empirical support that the cerebellum and its frontal white matter connections are implicated not only in the phonological loop of the verbal working memory, but more specifically correlated with auditory attention span.

### Limitations

Although our study provided new data concerning cerebellum lateralization, it also has some limitations that warrant mentioning. First, the findings should be interpreted carefully due to by heterogeneity of the patients’ group. The cerebellar lesion group included not only patients with tumors of the cerebellum, but also patients after a stroke of this brain structure or with vascular malformations. Moreover, cerebellar tumors that patients were diagnosed with differed significantly in terms of histology and WHO grade classification; therefore, they may vary in the risk of cognitive functioning impairment. Additionally, it is well established that emotional states affect cognitive functioning not only in individuals who undergo brain surgery, but also in controls who are treated for a spine degenerative disease.

A serious limitation of our research is the use of screening tests to assess cognitive functions, and although they are short, “patient-friendly” and have sufficient sensitivity, it is advisable to exercise great caution when formulating final and radical conclusions about the presence of cognitive disorders in a studied group of patients.

Future studies would benefit from including assessment of participants’ emotional state and analysis of their impact on cognitive processes in the study sample. Further research should also deal with more detailed examination of linguistic functions and their relationship with the right hemisphere, and also their possible associations with the disturbance of other cognitive processes to confirm the more global and more serious cognitive impairments related to the damage of the right cerebellum hemisphere.

## Conclusion

To sum up, our study tentatively confirmed that individuals with focal cerebellar lesions after tumor surgery or stroke differ in cognitive impairments pattern depending on the cerebellum lesion side. In line with our assumption, the analysis of individual cognitive domains indicated that patients with lesions localized in the left cerebellum hemisphere revealed decrease primarily in attention and visuospatial domains, whereas those with right-sided damage showed more global impairments which are beyond language difficulties.

## Data Availability Statement

The raw data supporting the conclusions of this article will be made available by the authors, without undue reservation.

## Ethics Statement

The studies involving human participants were reviewed and approved by Biomedical Ethics Committee of Jagiellonian University Medical College. The patients/participants provided their written informed consent to participate in this study.

## Author Contributions

AS-F designed the study, qualified patients for the group and conducted psychological tests, analyzed the data, and wrote the whole manuscript. KP revised the manuscript. JK analyzed statistically the data. AC revised the manuscript from the psychiatric perspective. AM helped with preparing the reference list. BB-K drafted the grant proposals that secured financing for the study. BK revised the manuscript from the neurosurgery perspective. All authors contributed to the article and approved the submitted version.

## Conflict of Interest

The authors declare that the research was conducted in the absence of any commercial or financial relationships that could be construed as a potential conflict of interest.

## Publisher’s Note

All claims expressed in this article are solely those of the authors and do not necessarily represent those of their affiliated organizations, or those of the publisher, the editors and the reviewers. Any product that may be evaluated in this article, or claim that may be made by its manufacturer, is not guaranteed or endorsed by the publisher.

## References

[B1] Ackermann H. Mathiak K. Ivry R. B. (2004). Temporal organization of “internal speech” as a basis for cerebellar modulation of cognitive functions. *Behav. Cogn. Neurosci. Rev.* 3 14–22. 10.1177/1534582304263251 15191639

[B2] Ackermann H. Mathiak K. Riecker A. (2007). The contribution of the cerebellum to speech production and speech perception: clinical and functional imaging data. *Cerebellum* 6 202–213. 10.1080/14734220701266742 17786816

[B3] Ailion A. S. King T. Z. Roberts S. R. Tang B. Turner J. A. Conway C. M. (2020). Double dissociation of auditory attention span and visual attention in long-term survivors of childhood cerebellar tumor: a deterministic tractography study of the cerebellar-frontal and the superior longitudinal fasciculus pathways. *J. Int. Neuropsychol. Soc.* 26 939–953. 10.1017/S1355617720000417 32342828

[B4] Andreasen N. C. Nopoulos P. O’Leary D. S. Miller D. D. Wassink T. Flaum M. (1999). Defining the phenotype of schizophrenia: cognitive dysmetria and its neural mechanisms. *Biol. Psychiatry* 46 908–920. 10.1016/s0006-3223(99)00152-3 10509174

[B5] Arasanz C. P. Staines W. R. Schweizer T. A. (2012). Isolating a cerebellar contribution to rapid visual attention using transcranial magnetic stimulation. *Front. Behav. Neurosci.* 6:55. 10.3389/fnbeh.2012.00055 22936903 PMC3426766

[B6] Baier B. Müller N. G. Dieterich M. (2014). What part of the cerebellum contributes to a visuospatial working memory task? *Ann. Neurol.* 76 754–757. 10.1002/ana.24272 25220347

[B7] Baillieux H. De Smet H. J. Dobbeleir A. Paquier P. F. De Deyn P. P. Mariën P. (2010). Cognitive and affective disturbances following focal cerebellar damage in adults: a neuropsychological and SPECT study. *Cortex* 46 869–879. 10.1016/j.cortex.2009.09.002 19853848

[B8] Baillieux H. De Smet H. J. Lesage G. Paquier P. De Deyn P. P. Mariën P. (2006). Neurobehavioral alterations in an adolescent following posterior fossa tumor resection. *Cerebellum* 5 289–295. 10.1080/14734220601009606 17134992

[B9] Baillieux H. Vandervliet E. J. Manto M. Parizel P. M. De Deyn P. P. Mariën P. (2009). Developmental dyslexia and widespread activation across the cerebellar hemispheres. *Brain Lang.* 108 122–132. 10.1016/j.bandl.2008.10.001 18986695

[B10] Bartczak E. Marcinowicz E. Kochanowski J. (2011). Cognitive function disorders in cerebellum stroke versus crossed diaschisis – case report. *Aktualności. Neurol.* 11 18–22.

[B11] Bauer P. M. Hanson J. L. Pierson R. K. Davidson R. J. Pollak S. D. (2009). Cerebellar volume and cognitive functioning in children who experienced early deprivation. *Biol. Psychiatry* 66 1100–1106. 10.1016/j.biopsych.2009.06.014 19660739 PMC2878609

[B12] Beebe L. H. Tian L. Morris N. Goodwin A. Allen S. S. Kuldau J. (2005). Effects of exercise on mental and physical health parameters of persons with schizophrenia. *Issues Ment. Health Nurs.* 26 661–676. 10.1080/01612840590959551 16020076

[B13] Ben-Yehudah G. Guediche S. Fiez J. A. (2007). Cerebellar contributions to verbal working memory: beyond cognitive theory. *Cerebellum* 6 193–201. 10.1080/14734220701286195 17786815

[B14] Bernard J. A. Seidler R. D. Hassevoort K. M. Benson B. L. Welsh R. C. Wiggins J. L. (2012). Resting state cortico-cerebellar functional connectivity networks: a comparison of anatomical and self-organizing map approaches. *Front. Neuroanat.* 6:31. 10.3389/fnana.2012.00031 22907994 PMC3415673

[B15] Blancart R. F. G. García Escrig M. Navarré Gimeno A. (2011). Aphasia secondary to a left cerebellar infarction. *Neurologia* 26 56–58. 10.1016/j.nrl.2010.09.006 21163241

[B16] Botez-Marquard T. Léveillé J. Botez M. I. (1994). Neuropsychological functioning in unilateral cerebellar damage. *Can. J. Neurol. Sci.* 21 353–357. 10.1017/s0317167100040956 7874621

[B17] Brunamonti E. Chiricozzi F. R. Clausi S. Olivito G. Giusti M. A. Molinari M. (2014). Cerebellar damage impairs executive control and monitoring of movement generation. *PLoS One* 9:e85997. 10.1371/journal.pone.0085997 24465830 PMC3895022

[B18] Bruno D. Schurmann Vignaga S. (2019). Addenbrooke’s cognitive examination III in the diagnosis of dementia: a critical review. *Neuropsychiatr. Dis. Treat.* 15 441–447. 10.2147/NDT.S151253 30858702 PMC6387595

[B19] Bucker R. L. (2013). The cerebellum and cognitive function: 25 years of insight from anatomy and neuroimaging. *Neuron* 80 807–815. 10.1016/j.neuron.2013.10.044 24183029

[B20] Cherkil S. Panikar D. Soman D. K. (2017). Profiling cognitive deficits in intra-axial and extra-axial tumors using Addenbrooke’s cognitive examination as a screening tool: an Indian experience. *Asian J. Neurosurg.* 12 653–658. 10.4103/ajns.AJNS_34_15 29114278 PMC5652090

[B21] Clower D. M. West R. A. Lynch J. C. Strick P. L. (2001). The inferior parietal lobule is the target of output from the superior colliculus, hippocampus, and cerebellum. *J. Neurosci.* 21 6283–6291. 10.1523/JNEUROSCI.21-16-06283.2001 11487651 PMC6763148

[B22] Cook M. Murdoch B. Cahill L. Whelan B. M. (2004). Higher-level language deficits resulting from left primary cerebellar lesions. *Aphasiology* 18 771–784. 10.1080/02687030444000291

[B23] Daszkiewicz P. Maryniak A. Roszkowski M. Barszcz S. (2009). Long-term functional outcome of surgical treatment of juvenile pilocytic astrocytoma of the cerebellum in children. *Childs Nerv. Syst.* 25 855–860. 10.1007/s00381-009-0855-1 19418058

[B24] De Smet H. J. Engelborghs S. Paquier P. F. De Deyn P. P. Mariën P. (2011). Cerebellar-induced apraxic agraphia: a review and three new cases. *Brain Cogn.* 76 424–434. 10.1016/j.bandc.2010.12.006 21507544

[B25] Egner T. Hirsch J. (2005). Where memory meets attention: neural substrates of negative priming. *J. Cogn. Neurosci.* 17 1774–1784. 10.1162/089892905774589226 16269113

[B26] Engelborghs S. Mariën P. Martin J. J. De Deyn P. P. (1998). Functional anatomy, vascularisation and pathology of the human thalamus. *Acta Neurol. Belg.* 98 252–265. 9801706

[B27] Exner C. Boucsein K. Degner D. Irle E. Weniger G. (2004). Impaired emotional learning and reduced amygdala size in schizophrenia: a 3-month follow-up. *Schizophr. Res.* 71 493–503. 10.1016/j.schres.2004.02.023 15474920

[B28] Falkenstein M. Hielscher H. Dziobek I. Schwarzenau P. Hoormann J. Sunderman B. (2001). Action monitoring, error detection, and the basal ganglia: an ERP study. *Neuroreport* 12 157–161. 10.1097/00001756-200101220-00039 11201078

[B29] Fiez J. A. Petersen S. E. Cheney M. K. Raichle M. E. (1992). Impaired non-motor learning and error detection associated with cerebellar damage. A single case study. *Brain* 115(Pt 1) 155–178. 10.1093/brain/115.1.155 1559151

[B30] Figlus M. Kaczorowska B. Jaskólski D. J. Kępczyński Ł (2018). Von Hippel-Lindau syndrome – a case report [Von Hippel-Lindau syndrome – a case report]. *Pol. Merkur. Lekarski* 44 248–252. 29813043

[B31] Fink G. R. Marshall J. C. Shah N. J. Weiss P. H. Halligan P. W. Grosse-Ruyken M. (2000). Line bisection judgments implicate right parietal cortex and cerebellum as assessed by fMRI. *Neurology* 54 1324–1331. 10.1212/wnl.54.6.1324 10746605

[B32] Fliessbach K. Weber B. Trautner P. Dohmen T. Sunde U. Elger C. E. (2007). Social comparison affects reward-related brain activity in the human ventral striatum. *Science* 318 1305–1308. 10.1126/science.1145876 18033886

[B33] Frings M. Dimitrova A. Schorn C. F. Elles H. G. Hein-Kropp C. Gizewski E. R. (2006). Cerebellar involvement in verb generation: an fMRI study. *Neurosci. Lett.* 409 19–23. 10.1016/j.neulet.2006.08.058 17046160

[B34] Gottwald B. Wilde B. Mihajlovic Z. Mehdorn H. M. (2004). Evidence for distinct cognitive deficits after focal cerebellar lesions. *J. Neurol. Neurosurg. Psychiatry* 75 1524–1531. 10.1136/jnnp.2003.018093 15489381 PMC1738803

[B35] Grafman J. Litvan I. Massaquoi S. Stewart M. Sirigu A. Hallett M. (1992). Cognitive planning deficit in patients with cerebellar atrophy. *Neurology* 42 1493–1496. 10.1212/wnl.42.8.1493 1641142

[B36] Grimaldi G. Manto M. (2011). Topography of cerebellar deficits in humans. *Cerebellum* 2 336–351. 10.1007/s12311-011-0247-4 21240580

[B37] Guell X. Gabrieli J. Schmahmann J. D. (2018). Triple representation of language, working memory, social and emotion processing in the cerebellum: convergent evidence from task and seed-based resting-state fMRI analyses in a single large cohort. *Neuroimage* 172 437–449. 10.1016/j.neuroimage.2018.01.082 29408539 PMC5910233

[B38] Habas C. Guillevin R. Abanou A. (2011). Functional connectivity of the superior human temporal sulcus in the brain resting state at 3T. *Neuroradiology* 53 129–140. 10.1007/s00234-010-0775-5 20924756

[B39] Häberling I. S. Corballis M. C. (2016). Cerebellar asymmetry, cortical asymmetry and handedness: two independent networks. *Laterality* 21 397–414. 10.1080/1357650X.2015.1110161 26582534

[B40] Hautzel H. Mottaghy F. M. Specht K. Müller H. W. Krause B. J. (2009). Evidence of a modality-dependent role of the cerebellum in working memory? An fMRI study comparing verbal and abstract n-back tasks. *Neuroimage* 47 2073–2082. 10.1016/j.neuroimage.2009.06.005 19524048

[B41] Hodges J. R. Larner A. J. (2017). “Addenbrooke’s cognitive examinations: ACE, ACE-R, ACE-III, ACEapp, and M-ACE,” in *Cognitive Screening Instruments: A Practical Approach*, 2nd Edn. ed. Larner A. J. (Berlin: Springer), 109–137.

[B42] Hokkanen L. S. Kauranen V. Roine R. O. Salonen O. Kotila M. (2006). Subtle cognitive deficits after cerebellar infarcts. *Eur. J. Neurol.* 13 161–170. 10.1111/j.1468-1331.2006.01157.x 16490047

[B43] Hubrich-Ungureanu P. Kaemmerer N. Henn F. A. Braus D. F. (2002). Lateralized organization of the cerebellum in a silent verbal fluency task: a functional magnetic resonance imaging study in healthy volunteers. *Neurosci. Lett.* 319 91–94. 10.1016/s0304-3940(01)02566-6 11825678

[B44] Ito M. (2008). Control of mental activities by internal models in the cerebellum. *Nat. Rev. Neurosci.* 9 304–313. 10.1038/nrn2332 18319727

[B45] Jansen A. Flöel A. Van Randenborgh J. Konrad C. Rotte M. Förster A. F. (2005). Crossed cerebro-cerebellar language dominance. *Hum. Brain Mapp.* 24 165–172. 10.1002/hbm.20077 15486988 PMC6871732

[B46] Karaci R. Oztürk S. Ozbakir S. Cansaran N. (2008). Evaluation of language functions in acute cerebellar vascular diseases. *J. Stroke Cerebrovasc. Dis.* 17 251–256. 10.1016/j.jstrokecerebrovasdis.2008.02.009 18755402

[B47] Karatekin C. Lazareff J. A. Asarnow R. F. (2000). Relevance of the cerebellar hemispheres for executive functions. *Pediatr. Neurol.* 22 106–112. 10.1016/s0887-8994(99)00128-9 10738915

[B48] Kavaklioglu T. Guadalupe T. Zwiers M. Marquand A. F. Onnink M. Shumskaya E. (2017). Structural asymmetries of the human cerebellum in relation to cerebral cortical asymmetries and handedness. *Brain Struct. Funct.* 222 1611–1623. 10.1007/s00429-016-1295-9 27566607 PMC5326706

[B49] Kelly R. M. Strick P. L. (2003). Cerebellar loops with motor cortex and prefrontal cortex of a nonhuman primate. *J. Neurosci.* 23 8432–8444. 10.1523/JNEUROSCI.23-23-08432.2003 12968006 PMC6740694

[B50] Kerrigan K. Rooney A. Grant G. (2011). Measuring cognitive function in people with brain tumours using the Addenbrooke’s cognitive examination. *J. Neurol. Neurosurg. Psychiatry* 83:e1. 10.4103/ajns.ajns_34_15

[B51] Klein A. P. Ulmer J. L. Quinet S. A. Mathews V. Mark L. P. (2016). Nonmotor functions of the cerebellum: an introduction. *Am. J. Neuroradiol.* 37 1005–1009. 10.3174/ajnr.A4720 26939633 PMC7963530

[B52] Koziol L. F. Budding D. Andreasen N. D’Arrigo S. Bulgheroni S. Imamizu H. (2014). Consensus paper: the cerebellum’s role in movement and cognition. *Cerebellum* 13 151–177. 10.1007/s12311-013-0511-x 23996631 PMC4089997

[B53] Krienen F. M. Buckner R. L. (2009). Segregated fronto-cerebellar circuits revealed by intrinsic functional connectivity. *Cereb. Cortex* 19 2485–2497. 10.1093/cercor/bhp135 19592571 PMC2742600

[B54] Lees R. A. Hendry Ba K. Broomfield N. Stott D. Larner A. J. Quinn T. J. (2017). Cognitive assessment in stroke: feasibility and test properties using differing approaches to scoring of incomplete items. *Int. J. Geriatr. Psychiatry* 32 1072–1078. 10.1002/gps.4568 27526678

[B55] Leggio M. G. Solida A. Silveri M. C. Gainotti G. Molinari M. (1995). Verbal fluency impairments in patients with cerebellar lesions. *Soc. Neurosci. Abstr.* 21:917.

[B56] Leggio M. G. Tedesco A. M. Chiricozzi F. R. Clausi S. Orsini A. Molinari M. (2008). Cognitive sequencing impairment in patients with focal or atrophic cerebellar damage. *Brain* 131(Pt 5) 1332–1343. 10.1093/brain/awn040 18334535

[B57] Lesage E. Hansen P. C. Miall R. C. (2017). Right lateral cerebellum represents linguistic predictability. *J. Neurosci.* 37 6231–6241. 10.1523/JNEUROSCI.3203-16.2017 28546307 PMC5490062

[B58] Levisohn L. Cronin-Golomb A. Schmahmann J. D. (2000). Neuropsychological consequences of cerebellar tumour resection in children: cerebellar cognitive affective syndrome in a paediatric population. *Brain* 123(Pt 5) 1041–1050. 10.1093/brain/123.5.1041 10775548

[B59] Lezak M. D. Howieson D. B. Loring D. W. (2004). *Neuropsychological Assessment*, 4th Edn. New York, NY: Oxford University Press.

[B60] Mak M. Tyburski E. Madany Ł Sokołowski A. Samochowiec A. (2016). Executive function deficits in patients after cerebellar neurosurgery. *J. Int. Neuropsychol. Soc.* 22 47–57. 10.1017/S1355617715001174 26626541

[B61] Marian V. Blumenfeld H. K. Kaushanskaya M. (2007). The Language Experience and Proficiency Questionnaire (LEAP-Q): assessing language profiles in bilinguals and Multilinguals. *J. Speech Lang. Hear. Res.* 50 940–967. 10.1044/1092-4388(2007/067) 17675598

[B62] Mariën P. Ackermann H. Adamaszek M. Barwood C. H. Beaton A. Desmond J. (2014). Consensus paper: language and the cerebellum: an ongoing enigma. *Cerebellum* 13 386–410. 10.1007/s12311-013-0540-5 24318484 PMC4090012

[B63] Mariën P. Saerens J. Nanhoe R. Moens E. Nagels G. Pickut B. A. (1996). Cerebellar induced aphasia: case report of cerebellar induced prefrontal aphasic language phenomena supported by SPECT findings. *J. Neurol. Sci.* 144 34–43. 10.1016/s0022-510x(96)00059-7 8994102

[B64] Marvel C. L. Desmond J. E. (2012). From storage to manipulation: how the neural correlates of verbal working memory reflect varying demands on inner speech. *Brain Lang.* 120 42–51. 10.1016/j.bandl.2011.08.005 21889195 PMC3242899

[B65] Marvel C. L. Desmond J. E. (2010). The contributions of cerebro-cerebellar circuitry to executive verbal working memory. *Cortex* 46 880–895. 10.1016/j.cortex.2009.08.017 19811779 PMC2872048

[B66] Marvel C. L. Faulkner M. L. Strain E. C. Mintzer M. Z. Desmond J. E. (2012). An fMRI investigation of cerebellar function during verbal working memory in methadone maintenance patients. *Cerebellum* 11 300–310. 10.1007/s12311-011-0311-0 21892700 PMC3248617

[B67] Mathuranath P. S. Nestor P. J. Berrios G. E. Rakowicz W. Hodges J. R. (2000). A brief cognitive test battery to differentiate Alzheimer’s disease and frontotemporal dementia. *Neurology* 55 1613–1620. 10.1212/01.wnl.0000434309.85312.19 11113213

[B68] Misciagna S. Iuvone L. Mariotti P. Silveri M. C. (2010). Verbal short-term memory and cerebellum: evidence from a patient with congenital cerebellar vermis hypoplasia. *Neurocase* 16 119–124. 10.1080/13554790903329158 19927261

[B69] Miyake A. Friedman N. P. Emerson M. J. Witzki A. H. Howerter A. Wager T. D. (2000). The unity and diversity of executive functions and their contributions to complex “Frontal Lobe” tasks: a latent variable analysis. *Cogn. Psychol.* 41 49–100. 10.1006/cogp.1999.0734 10945922

[B70] Molinari M. Petrosini L. Misciagna S. Leggio M. G. (2004). Visuospatial abilities in cerebellar disorders. *J. Neurol. Neurosurg. Psychiatry* 75 235–240. 14742596 PMC1738892

[B71] O’Hare E. D. Lu L. H. Houston S. M. Bookheimer S. Y. Sowell E. R. (2008). Neurodevelopmental changes in verbal working memory load-dependency: an fMRI investigation. *Neuroimage* 42 1678–1685. 10.1016/j.neuroimage.2008.05.057 18586110 PMC2570587

[B72] Paulesu E. Frith C. D. Frackowiak R. S. (1993). The neural correlates of the verbal component of working memory. *Nature* 362 342–345. 10.1038/362342a0 8455719

[B73] Perez M. Amayra I. Lazaro E. García M. Martínez O. Caballero P. (2020). Intrusion errors during verbal fluency task in amyotrophic lateral sclerosis. *PLoS One* 15:e0233349. 10.1371/journal.pone.0233349 32469951 PMC7259757

[B74] Petersen S. E. Fox P. T. Posner M. I. Mintun M. Raichle M. E. (1989). Positron emission tomographic studies of the processing of singe words. *J. Cogn. Neurosci.* 1 153–170. 10.1162/jocn.1989.1.2.153 23968463

[B75] Ramnani N. (2006). The primate cortico-cerebellar system: anatomy and function. *Nat. Rev. Neurosci.* 7 511–522. 10.1038/nrn1953 16791141

[B76] Reitan R. Wolfson D. (1993). *The Halstead-Reitan Neuropsychological Test Battery: Theory and Clinical Interpretation.* Tucson, AZ: Neuropsychology Press.

[B77] Richter S. Gerwig M. Aslan B. Wilhelm H. Schoch B. Dimitrova A. (2007). Cognitive functions in patients with MR-defined chronic focal cerebellar lesions. *J. Neurol.* 254 1193–1203. 10.1007/s00415-006-0500-9 17380238

[B78] Riva D. Cazzaniga F. Esposito S. Bulgheroni S. (2013). Executive functions and cerebellar development in children. *Appl. Neuropsychol. Child* 2 97–103. 10.1080/21622965.2013.791092 23745837

[B79] Riva D. Giorgi C. (2000). The neurodevelopmental price of survival in children with malignant brain tumours. *Childs Nerv. Syst.* 16 751–754. 10.1007/s003810000332 11151727

[B80] Rouleau I. Imbault H. Laframboise M. Bédard M. A. (2001). Pattern of intrusions in verbal recall: comparison of Alzheimer’s disease, Parkinson’s disease, and frontal lobe dementia. *Brain Cogn.* 46 244–249. 10.1016/s0278-2626(01)80076-2 11527341

[B81] Salman M. S. Tsai P. (2016). The role of the pediatric cerebellum in motor functions, cognition, and behavior: a clinical perspective. *Neuroimaging Clin. North Am.* 26 317–329. 10.1016/j.nic.2016.03.003 27423796 PMC4948592

[B82] Schmahmann J. D. (1991). An emerging concept. The cerebellar contribution to higher function. *Arch. Neurol.* 48 1178–1187. 10.1001/archneur.1991.00530230086029 1953406

[B83] Schmahmann J. D. (1996). From movement to thought: anatomic substrates of the cerebellar contribution to cognitive processing. *Hum. Brain Mapp.* 4 174–198. 10.1002/(SICI)1097-0193(1996)4:3<174::AID-HBM3>3.0.CO;2-0 20408197

[B84] Schmahmann J. (2001). The cerebrocerebellar system: anatomic substrates of the cerebellar contribution to cognition and emotion. *Int. Rev. Psychiatry* 13 247–260. 10.1080/09540260120082092

[B85] Schmahmann J. D. (2010). The role of the cerebellum in cognition and emotion: personal reflections since 1982 on the dysmetria of thought hypothesis, and its historical evolution from theory to therapy. *Neuropsychol. Rev.* 20 236–260. 10.1007/s11065-010-9142-x 20821056

[B86] Schmahmann J. D. Pandya D. N. (1997). The cerebrocerebellar system. *Int. Rev. Neurobiol.* 41 31–60. 10.1016/s0074-7742(08)60346-3 9378595

[B87] Schmahmann J. D. Sherman J. C. (1998). The cerebellar cognitive affective syndrome. *Brain* 121(Pt 4) 561–579. 10.1093/brain/121.4.561 9577385

[B88] Scott R. B. Stoodley C. J. Anslow P. Paul C. Stein J. F. Sugden E. M. (2001). Lateralized cognitive deficits in children following cerebellar lesions. *Dev. Med. Child Neurol.* 43 685–691. 10.1017/s0012162201001232 11665825

[B89] Starowicz-Filip A. Chrobak A. A. Kwiatkowski S. Milczarek O. Rajtar-Zembaty A. (2020). Cerebellar lesions after low-grade tumor resection can induce memory impairments in children, similar to that observed in patients with frontal lesions. *Child Neuropsychol.* 26 388–408. 10.1080/09297049.2019.1657391 31451041

[B90] Starowicz-Filip A. Chrobak A. A. Milczarek O. Kwiatkowski S. (2017). The visuospatial functions in children after cerebellar low-grade astrocytoma surgery: a contribution to the pediatric neuropsychology of the cerebellum. *J. Neuropsychol.* 11 201–221. 10.1111/jnp.12093 26638981

[B91] Starowicz-Filip A. Prochwicz K. Kłosowska J. Chrobak A. A. Krzyżewski R. Myszka A. (2021). Is Addenbrooke’s cognitive examination III sensitive enough to detect cognitive dysfunctions in patients with focal cerebellar lesions? *Arch. Clin. Neuropsychol.* acab045. Advance online publication 10.1093/arclin/acab045 34128041

[B92] Steinlin M. Imfeld S. Zulauf P. Boltshauser E. Lovblad K. O. Luthy A. R. (2003). Neuropsychological long-term sequelae after posterior fossa tumour resection during childhood. *Brain* 126 1998–2008. 12876140 10.1093/brain/awg195

[B93] Stoodley C. J. MacMore J. P. Makris N. Sherman J. C. Schmahmann J. D. (2016). Location of lesion determines motor vs. cognitive consequences in patients with cerebellar stroke. *Neuroimage Clin.* 12 765–775. 10.1016/j.nicl.2016.10.013 27812503 PMC5079414

[B94] Stoodley C. J. Schmahmann J. D. (2009). Functional topography in the human cerebellum: a meta-analysis of neuroimaging studies. *Neuroimage* 44 489–501. 10.1016/j.neuroimage.2008.08.039 18835452

[B95] Stoodley C. J. Valera E. M. Schmahmann J. D. (2010). An fMRI study of intra-individual functional topography in the human cerebellum. *Behav. Neurol.* 23 65–79. 10.3233/BEN-2010-0268 20714062 PMC3776583

[B96] Tavano A. Grasso R. Gagliardi C. Triulzi F. Bresolin N. Fabbro F. (2007). Disorders of cognitive and affective development in cerebellar malformations. *Brain* 130(Pt 10) 2646–2660. 10.1093/brain/awm201 17872929

[B97] Timmann D. Daum I. (2007). Cerebellar contributions to cognitive functions: a progress report after two decades of research. *Cerebellum* 6 159–162. 10.1080/14734220701496448 17786810

[B98] Tröster A. I. Fields J. A. Testa J. A. Paul R. H. Blanco C. R. Hames K. A. (1998). Cortical and subcortical influences on clustering and switching in the performance of verbal fluency tasks. *Neuropsychologia* 36 295–304. 10.1016/s0028-3932(97)00153-x 9665640

[B99] Ullsperger M. von Cramon D. Y. (2006). The role of intact frontostriatal circuits in error processing. *J. Cogn. Neurosci.* 18 651–664. 10.1162/jocn.2006.18.4.651 16768367

[B100] Ziemus B. Baumann O. Luerding R. Schlosser R. Schuierer G. Bogdahn U. (2007). Impaired working-memory after cerebellar infarcts paralleled by changes in BOLD signal of a cortico-cerebellar circuit. *Neuropsychologia* 45 2016–2024. 10.1016/j.neuropsychologia.2007.02.012 17379262

